# *Lactobacillus johnsonii* HL79 modulates the microbiota-gut-brain axis to protect cognitive function in mice chronically exposed to high altitude

**DOI:** 10.3389/fmicb.2025.1561400

**Published:** 2025-03-07

**Authors:** Zhifang Zhao, Xufei Zhang, Ning Sun, Lixiao Duan, Jinge Xin, Hao Li, Xueqin Ni, Hesong Wang, Hailin Ma, Yang Bai

**Affiliations:** ^1^Guangdong Provincial Key Laboratory of Gastroenterology, Department of Gastroenterology, Institute of Gastroenterology of Guangdong Province, Nanfang Hospital, Southern Medical University, Guangzhou, China; ^2^Department of Gastroenterology, National Institution of Drug Clinical Trial, Guizhou Provincial People's Hospital, Medical College of Guizhou University, Guiyang, China; ^3^Key Laboratory of High Altitudes Brain, Science and Environmental Acclimation, Tibet University, Lhasa, China; ^4^Plateau Brain Science Research Center, Tibet University, Lhasa, China; ^5^Tibet Autonomous Region Psychological Society, Lhasa, China; ^6^Animal Microecology Institute, College of Veterinary Medicine, Sichuan Agricultural University, Chengdu, China; ^7^Baiyun Branch, Nanfang Hospital, Southern Medical University, Guangzhou, China

**Keywords:** high-altitude exposure, microbiome-gut-brain axis, probiotics, *Lactobacillus johnsonii*, working memory

## Abstract

**Introduction:**

High-altitude environments have significant effects on brain function, particularly a decline in cognitive function, due to insufficient oxygen supply. The microbiome-gut-brain axis (MGBA) plays an important role in regulating cognitive function, but its specific mechanism of action in high-altitude environments is unclear. Therefore, the aim of this study was to investigate whether the probiotic *Lactobacillus johnsonii* HL79 could alleviate high altitude-induced cognitive dysfunction in mice by modulating the gut microbiota.

**Methods and results:**

Sixty C57BL/6 mice aged 8 weeks were randomly divided into four groups: control, high altitude exposure (HA), HL79-treated (P), and high altitude exposure plus HL79-treated (HAP). the HA and HAP groups were exposed to a low-pressure oxygen chamber at a simulated altitude of 3,500–4,000 m for 20 weeks, while the Control and P groups were maintained at the normal barometric pressure level. Probiotic HL79 was given daily by gavage in the P and HAP groups, while saline gavage was given daily in the other two groups. The cognitive functions of the mice were assessed by new object recognition test and elevated plus maze test. The results showed that HL79 treatment significantly improved the working memory abilities of high altitude exposed mice. In addition, HL79 treatment improved antioxidant capacity, decreased malondialdehyde (MDA) content, and increased superoxide dismutase (SOD) and catalase (CAT) activities in serum and whole brain tissue. Gut microbiota analysis showed that HL79 was able to modulate the structure of gut microbiota and increase the relative abundance of beneficial flora in high altitude environment.

**Conclusion:**

*Lactobacillus johnsonii* HL79 significantly ameliorated cognitive dysfunction in high altitude-exposed mice by modulating the gut microbiota and antioxidant capacity, further confirming the important role of MGBA in high altitude environment.

## Introduction

High altitude has multiple effects on human physiological function due to the scarcity of oxygen. Among these are significant challenges to brain function, mainly due to the reduced supply of oxygen to the brain under hypoxic conditions. Hypoxia-inducible factors (HIF) play a crucial role in adapting the brain to low oxygen levels by influencing neuronal survival, metabolism, synaptic formation, and plasticity. These molecular changes lead to cognitive decline, including impairments in memory, attention, and reaction speed (Aboouf et al., 2023). Prolonged exposure to high altitudes causes brain gray matter atrophy, slowed neural transmission, and cognitive decline, particularly affecting inhibitory control, attention, and memory (Chen et al., 2023). Brain imaging techniques, such as Functional Magnetic Resonance Imaging and Event-Related Potential, reveal structural changes in the brain associated with these impairments, which worsen with longer exposure times. Additionally, high-altitude environments also affect gut function, significantly reducing the expression of tight junction proteins such as claudin-1, occludin, and ZO-1, leading to increased gut permeability (Cheng et al., 2024). Studies have shown that hypoxia-induced inflammatory responses and mucosal barrier damage weaken the gut’s barrier function, making it more vulnerable to external stimuli (Cheng et al., 2022). Furthermore, high-altitude environments induce marked alterations in the gut microbiota. Analysis of the gut microbiota in high-altitude populations reveals significant changes in composition and diversity under hypoxic conditions, with an increase in specific microbial populations such as “Blautia A,” which helps maintain microbial community stability and aids in human adaptation to low-oxygen environments (Cheng et al., 2022).

The gut-brain axis (GBA) refers to the bidirectional communication network between the gut microbiota, the enteric nervous system, and the central nervous system (CNS) (Góralczyk-Bińkowska et al., 2022; Kraimi et al., 2024). In recent years, research on the GBA has advanced, showing that it involves not only physical connections between the gut and brain but also complex interactions mediated by the vagus nerve, immune responses, endocrine pathways, and microbial metabolites (Person and Keefer, 2021; Rutsch et al., 2020; Kasarello et al., 2023). Based on the previous studies, medical researchers have further developed the concept of “microbiota-gut-brain axis (MGBA),” whose bidirectional communication mechanism encompasses neurological, endocrine and immune pathways, and has a significant impact on the pathophysiological processes of various neurological diseases (Cryan et al., 2019). Gut microbiota can influence host physiology and mental health, playing a key role in regulating mood, behavior, and cognitive function. Disruptions in the gut microbiome have been linked to mental disorders such as depression, anxiety, and autism spectrum disorder (ASD) (Liu et al., 2022; Xiong et al., 2023; Romano et al., 2023). Moreover, with increasing knowledge of MGBA complexity, certain probiotics, known as “psychobiotics,” have been found to improve brain health by modulating the composition and function of the gut microbiome (Di Chiano et al., 2024; Ķimse et al., 2024). These psychobiotics not only enhance gut health but also improve brain function by influencing neurotransmitter secretion, boosting the immune system, and reducing inflammation, thus benefiting the host’s mental health (Del Toro-Barbosa et al., 2020; Binda et al., 2024). Several studies have found that specific probiotic strains can influence the metabolites of the gut flora by modulating the production of short-chain fatty acids (SCFAs), which in turn can positively affect brain function (Yang et al., 2020). In addition, probiotics may also have a modulatory effect on cognitive function by modulating the activity of the enteric nervous system and influencing vagal signaling (Lekchand Dasriya et al., 2022). It has also been suggested that probiotics can reduce the production of inflammatory factors by modulating the composition of the gut flora, thereby improving the neuroinflammatory state associated with cognitive function (Suresh et al., 2024). Studies have shown that *Lactobacillus johnsonii* BS15 can alleviate stress-induced memory dysfunction in mice by modulating gut inflammation and permeability. This probiotic strain reduces stress-induced gut damage, enhances neurotransmitter levels in the brain, and improves synaptic plasticity in the hippocampus (Wang et al., 2021). Similarly, *Lactobacillus plantarum* C29 has been found to alleviate memory impairment in Alzheimer’s disease model mice by regulating the gut microbiota and microglial activation, revealing its potential in preventing neurodegenerative diseases (Lee et al., 2018). Furthermore, “*Lactobacillus reuteri*” has demonstrated the ability to improve social behavior deficits in mouse models of ASD, highlighting its positive effects on brain social behavior by regulating the gut microbiota (Sgritta et al., 2019). These findings suggest that probiotic has the potential to attenuate stress-induced brain damage and improve neurocognitive function through the gut-brain axis.

Given the negative effects of high altitude exposure on gut and brain function, the microbiota-gut-brain axis may be a potential therapeutic strategy to mitigate high altitude-induced cognitive decline and stress. Previous studies by our research group have shown that disruption of the gut microbiota by antibiotics exacerbates spatial and working memory deficits induced by short-term (14-day) high altitude exposure in mice, which in turn impairs hippocampal and prefrontal cortex function (Zhao et al., 2022; Zhang et al., 2023). For example, *Lactobacillus johnsonii* was able to alleviate restraint stress-induced memory dysfunction in mice by modulating intestinal inflammation and permeability (Wang et al., 2021). This probiotic enhanced cognitive function by improving gut barrier function and reducing inflammatory factor levels, suggesting that it may attenuate stress-related brain damage through the gut-brain axis. In addition, it was found that *Lactobacillus johnsonii* BS15 prevented psychological stress-induced memory dysfunction in mice by improving the intestinal environment and strengthening the intestinal barrier, ultimately reducing inflammation and enhancing cognitive performance (Wang et al., 2020). According to our previous studies, *Lactobacillus johnsonii* has shown promising effects in the prevention and treatment of NAFLD and in the alleviation of alcohol-induced memory impairment by modulating the gut microbiome (Sun et al., 2020; Sun et al., 2022). In addition, “*Lactobacillus johnsonii*” YH1136 has been shown to play a key role in attenuating kidney and intestinal damage induced by high fluoride exposure in our previous studies (Xin et al., 2021; Wan et al., 2022). These findings suggest that *Lactobacillus johnsonii* may improve cognitive and memory functions by modulating the gut-brain axis and play a positive role in reducing stress-induced brain damage.

In this study, we investigated whether *Lactobacillus johnsonii* HL79 (CCTCC AB 2025192), isolated from the feces of a healthy Tibetan girl in Nagchu, Tibet Autonomous Region, could alleviate altitude-induced cognitive decline by modulating the gut microbiota. We assessed cognitive function by evaluating behavioral performance in mice. We also measured serum and whole-brain oxidative stress levels and used high-throughput sequencing to compare gut microbiome composition. This study was designed to address the following scientific questions: (1) Can HL79 alleviate cognitive behavioral decline caused by chronic high altitude exposure? (2) What are the mechanisms behind its effects on typical behavioral responses?

## Materials and methods

### Bacteria preparation and animal treatments

*Lactobacillus johnsonii* HL79 was maintained in de Man, Rogosa and Sharpe (MRS, QDRS Biotec, Qingdao, Shandong, China) broth under anaerobic environment at 37°C for 36 h. Heterotrophic plate count was used to evaluate the amount of bacterial cells. After collection, the bacterial cells were washed with saline and then suspended at pH 7.0 in phosphate buffered saline (PBS) at a concentration of 1 × 10^9^ cfu *Lactobacillus johnsonii* HL79/mL.

A total of 60 male C57BL/6 mice (8 weeks old) were randomly divided into four groups (*n* = 15 per group): (1) Control group (Control), (2) High Altitude group (HA), (3) HL79 group (P), and (4) High Altitude + HL79 group (HAP). The air pressure level for the control and P groups were maintained at 94.5 kPa, while the HA and HAP groups were exposed to a low-pressure oxygen chamber simulating an altitude of 3,500–4,000 m for 20 weeks, with the air pressure set to 60–65 kPa. Mice in the P and HAP groups were given probiotic HL79 (0.2 mL of 1×10^9^ CFU/mL suspension), and the remaining two groups were given saline for 140 experimental days. All animal experiments were conducted in accordance with the guidelines for the feeding and use of experimental animals and approved by the Institutional Animal Care and Use Committee of the Sichuan Agricultural University (approval number: SYXKchuan2019-187) and the Animal Ethics Committee of Guizhou Provincial People’s Hospital (approval number: Guizhou Provincial People’s Hospital Ethical Review (Animal) 2023-009). All animals were kept in a room with a controlled temperature (22 ± 2°C) and a 12/12-h light/dark cycle (dark period: 7 p.m. to 7 a.m.). During the experiment, the body weight and overall health of the mice were monitored weekly to ensure that environmental and treatment conditions did not adversely affect their well-being. Additionally, the cages were maintained in a pathogen-free environment to prevent any external factors from influencing the outcomes of the study.

### Study design and sample collection

**Figure 1:**
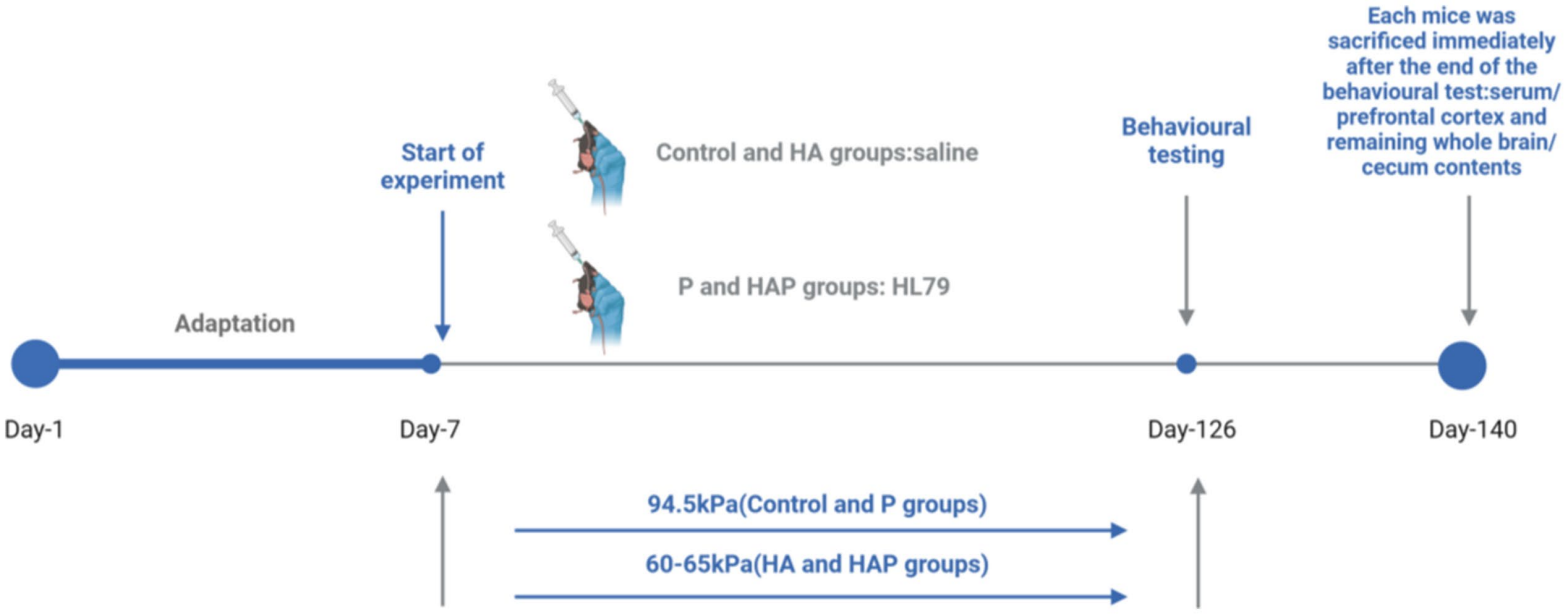
Flowchart of the whole animal experiment.

The study lasted for 20 weeks, during which the mice underwent behavioral testing starting in week 18. Tests included the Novel Object Recognition test to evaluate working memory and the Elevated Plus Maze to evaluate anxiety-like behavior. The behavioral tests were conducted in a controlled room with constant temperature (22 ± 2°C) and lighting conditions, ensuring minimal external stressors on the animals. Each test was conducted during the light phase of the 12-h light/dark cycle. At the end of the behavioral tests, the mice were immediately sacrificed by cervical dislocation, according to the guidelines of the animal care facility. Blood samples were collected via cardiac puncture and immediately placed on ice for subsequent centrifugation. Serum was stored at −20°C for later biochemical assays. The prefrontal cortex and remaining whole brain were immediately removed, and these samples were washed in ice-cold sterile saline, frozen in liquid nitrogen, and then stored at −80°C. In addition, the cecum contents of each mouse were collected and frozen at −20°C for microbiota analysis using 16S rRNA gene sequencing to assess gut microbiome composition. A flowchart of the animal experiments is shown in Figure 1.

## Behavioral tests

### Novel object recognition test

**Figure 2:**
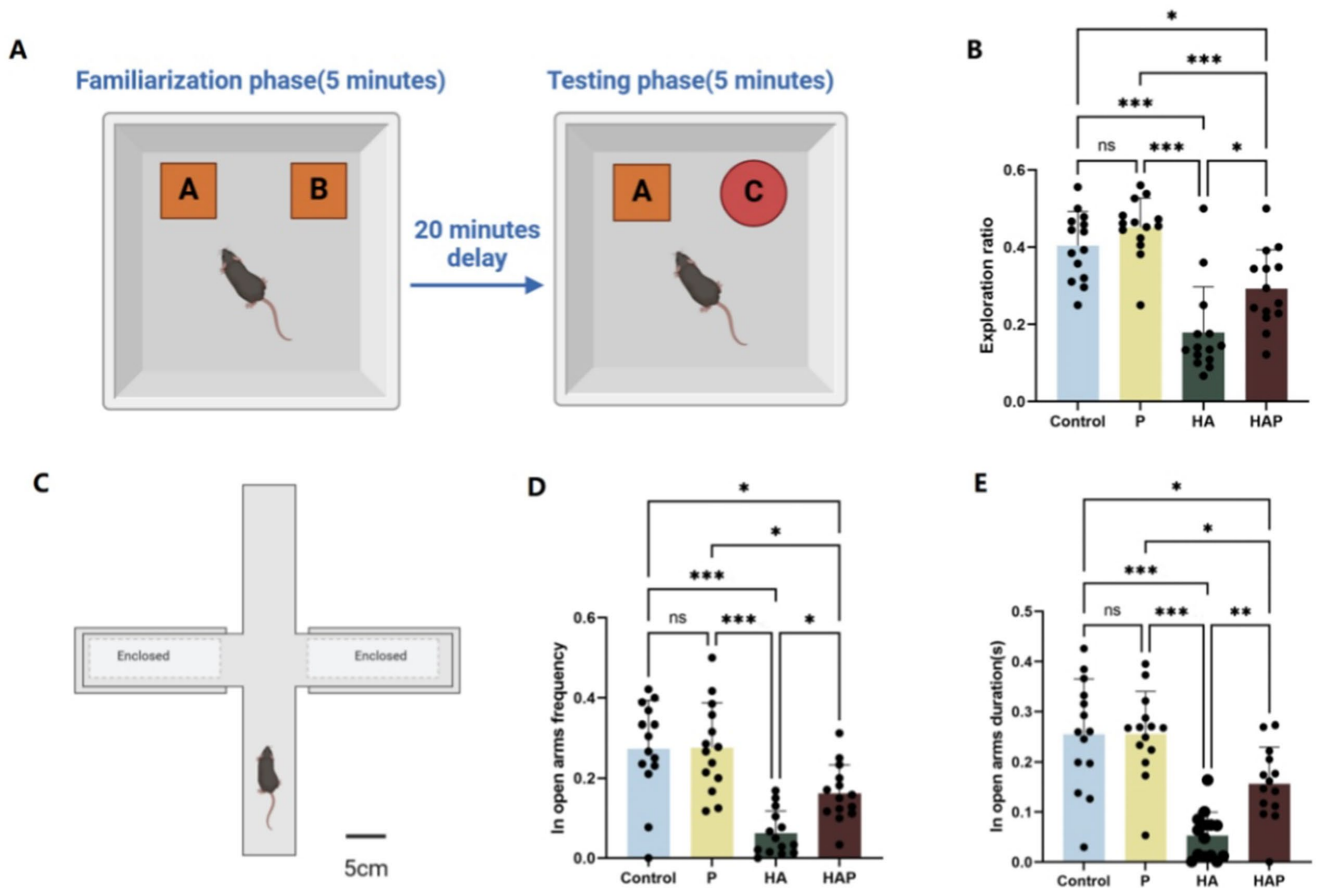
**(A)** Schematic diagram of the New Object Recognition test, **(B)** the exploration ratio in the New Object Recognition test. **(C)** Schematic diagram of the Elevated Plus Maze, **(D)** the open-arm frequency, and **(E)** the open-arm duration in the Elevated Plus Maze. Data are displayed with the mean ± SD (*n* = 14). Significant difference between groups is expressed on the basis of one-way ANOVA statistical analysis followed by LSD test (*p < 0.05). The “ns” means there is no significant difference between groups (*p* > 0.05); *difference is significant at the 0.05 level (*p* < 0.05); **difference is significant at the 0.01 level (*p* < 0.01); ***difference is significant at the 0.001 level (*p* < 0.001).

The Novel Object Recognition (NOR) test was used to evaluate recognition memory in mice, leveraging their instinct to explore unfamiliar objects. The experiment was carried out in a 40 × 40 × 45 cm open-field arena. On the day prior to testing, mice were habituated to the empty arena for 10 min. During the familiarization phase, two identical objects were placed in opposite corners of the arena, allowing mice to explore for 5 min. After a 20-min interval, one of the original objects was replaced with a novel one, and mice were given an additional 5 min to explore. Recognition memory was assessed by calculating the ratio of time spent exploring the new object relative to the familiar one. Increased exploration of the novel object indicated better memory retention. The results were statistically analyzed to evaluate differences in recognition performance between experimental groups. The experimental diagram is shown in Figure 2A.

### Elevated Plus Maze

The Elevated Plus Maze (EPM) was utilized to assess anxiety-like behavior in mice. The apparatus consisted of two opposing open arms (30 cm × 5 cm) and two enclosed arms (30 cm × 5 cm × 15 cm) arranged in a cross configuration, elevated 50 cm above the ground. Mice were individually placed in the center of the maze, facing an open arm, and allowed to explore freely for 5 min. During the test, the time spent in the open arms and the number of entries into the open arms were recorded. Open arm time and entries are inversely correlated with anxiety levels, as mice typically avoid open, elevated areas due to fear of exposure. Conversely, increased time and entries in the open arms indicate reduced anxiety-like behavior. The EPM test was conducted under controlled conditions, with ambient temperature and lighting kept constant to minimize external influences on behavior. Each test was performed during the light phase of the 12-h light/dark cycle. Data were analyzed to compare anxiety levels between experimental groups, and results were expressed as mean ± standard deviation. The experimental diagram is shown in Figure 2C.

### Biochemical analysis

Biochemical assays were performed to assess oxidative stress and antioxidant enzyme activities in the collected prefrontal cortex tissues. The activities of Total antioxidant capacity (T-AOC), superoxide dismutase (SOD), and catalase (CAT) were measured using commercial kits, following the manufacturer’s protocols. Additionally, malondialdehyde (MDA) levels were analyzed to evaluate lipid peroxidation as a marker of oxidative damage. All measurements were conducted in triplicate, and the results were expressed as mean ± standard deviation. Group comparisons were performed using appropriate statistical tests to identify significant differences in antioxidant capacity and oxidative damage between experimental groups. These biochemical parameters were used to infer the relationship between treatment interventions and oxidative stress responses.

### Bioinformatic analysis of gut microbiota

Total DNA samples from mouse cecum contents were extracted using the E.Z. N.A. TM Fecal DNA Extraction Kit (OMEGA Bio-Tek, Norcross, GA, United States) in a final elution volume of 100 μL. The integrity and quality of the extracted DNA were determined by using a NanoDrop NC2000 spectrophotometer (Thermo Fisher Scientific, Waltham, MA, United States) and agarose gel electrophoresis, respectively. DNA samples were sent to Shanghai Personal Biotechnology Co. Ltd. Specific barcodes were inserted into the amplification primers, and the bacterial 16S rRNA gene was amplified by PCR and detected using the Illumina MiSeq sequencing platform and a fragment of the V3-V4 highly variable region 2 × 250 bp from the opposite end. The primer sequences were 338F (5′-ACTCCTACGGGGGAGGAGCA-3′) and 806R (5′-GGACTachVGGGGTWTCTAAT-3′). PCR amplification system: Fast Pfu DNA polymerase (5 U/μL) 0.25 μL, dNTPs (2.5 mM) 2 μL, buffer (5 ×) 5 μL, DNA template 1 μL, dNTPs (2.5 mM) 2 μL. l, DNA template 1 μL, ddH2O 14.75 μL forward and reverse primers (10 μM) 1 μL. The amplification program was set as follows: initial denaturation at 98°C for 5 min, 25 cycles; denaturation at 98°C for 30 s; annealing at 53°C for 30 s; extension at 72°C for 45 s; and extension at 72°C for 5 s. The amplification was performed using the Quant-iT PicoGreen dsDNA Assay Kit (Invitrogen, Carlsbad, CA, United States) and Vazyme VAHTSTM DNA Clean Beads (Vazyme, Nanjing, China) were used to quantify and purify PCR amplicons, respectively.

Referring to the official tutorials, the raw data were bioinformatically analyzed using the QIIME2 (Bolyen et al., 2019) tool. Using the feature-classifier plugin (Price et al., 2010) based on the Navier Bayes classifier and the Greengenes13_8 database for the abundance table of the species were labelled. Downstream analyses were mainly performed using Rstudio software (V3.1.2). Alpha diversity indices (number of species observed and Shannon diversity) were calculated with ASV abundance. For in-depth analyses,we selected samples each group in which at least 20% of the ASVs were present and where the sum of the relative abundances exceeded 2.5%, The filtered ASV sequence counts were subjected to a normalisation step using the TMM method in the edgeR package (Robinson and Oshlack, 2010). Gut community structure was assessed using the principal coordinate analysis (PCoA) based on Bray–Curtis dissimilarity (McMurdie and Holmes, 2013). In addition, a permutation multivariate analysis of variance (peromova) was used to assess differences in gut microbial communities across treatments using the adonis method. Microbial taxa from different treatments were clustered at the phylum and family level. In addition, we used indicator species analyses to obtain pointwise bicolumn correlation coefficients (r) for positive correlation of ASV with one or more treatments. A filter threshold of *p* < 0.05 was set to identify key species and visualize network diagrams.

According to the relative abundance threshold of different species, the rare or rich species are divided into different groups: (1) Rare taxa (RT), which is ≤0.1% of the relative abundance in the all samples. (2) Abundant taxa (AT), all of which are ≥1% of the relative abundance of all samples. (3) The Moderate taxa (MT), which is >0.1 and < 1% of the relative abundance in all samples. (4) Conditionally rare taxa (CRT), which is <1%in the all samples, and only <0.1 percent of the relative abundance of the partial samples. (5) Conditionally abundant taxa (CAT), all of which is >0.1% in the all samples, and only >1% of the relative abundance in the partial samples. (6) Conditionally rare or abundant taxa (CRAT), the abundance span from rare (minimum abundance of ≤0.1%) to the rich (highest abundance ≥1%) of the relative abundance.

To filter the rare or abundant taxa that play an important role in high altitude environment and *Lactobacillus johnsonii* HL79 preventing working memory impairment, we used different analytical methods to determine key species from rare or abundant taxa. At first, we calculated the specificity and occupancy of all ASVs for each microbial species, selected the characteristic species of different grouped taxa, and plotted specificity–occupancy diagrams to identify potential key species (specificity and occupancy >0.7).

Subsequently, we chose an interaction computational method suitable for the characteristics of high-throughput histological data. Molecular ecological network analysis (MENA) was used to elucidate the species co-occurrence of microbial communities under different treatments, determine their responses to environmental changes, and construct microbial interspecies interaction networks. Based on the cohesive and topological properties of the network, network nodes (abundant species) with properties such as the maximum node degree and maximum mediator number were defined as important nodes for maintaining network structure stability, whose absence may lead to decomposition of the network module and network.

### RNA-seq analysis

Total RNA was extracted from each group using a Total RNA Isolation Kit (Omega Inc., Norcross, Georgia, United States) according to the manufacturer’s instructions. The initial RNA for library construction is immediately interrupted in the Fragmentation Buffer. Using fragmented mRNA as a template, the first strand of cDNA was synthesized under the action of random oligonucleotide primers. Subsequently, the second strand of cDNA was synthesized using dNTPs as raw materials in the DNA polymerase I system. After purification, the double stranded cDNA was subjected to terminal repair, A-tail addition, and sequencing adapter ligation, and the final library was obtained by PCR amplification. Different samples were sequenced using Illumina to obtain raw data for FASTQ. To ensure the quality of data analysis, quality control is applied to the raw data, including removing connectors and filtering out low-quality data. Construct a reference genome index using HISAT2 (v2.0.5) and compare the filtered reads with the reference genome. Subsequently, feature counts were used to calculate the reading of each gene, and the FPKM of each gene and the reading mapped to that gene were calculated based on its length. By using PCA method, the clustering degree of overall gene expression within four groups and the distribution trend of samples between groups can be observed. In order to identify differentially expressed genes among different groups, edgeR software was used to analyze the differential expression between different groups. It was found that genes with log2foldchange > 1 and *p* < 0.05 were assigned as differentially expressed genes. By using clusterProfiler software to achieve GO enrichment analysis of differentially expressed genes, advanced functions and effects of biological systems can be understood from molecular level information, especially large-scale molecular datasets generated by genome sequencing and other high-throughput databases. Subsequently, the gene list and number for each GO term were calculated using clusterProfiler software. By using the hypergeometric distribution method, GO terms with significantly enriched genes compared to the entire genome background can be identified to determine the main biological functions of differentially expressed genes.

For bioinformatics analyses, we chose QIIME2 (Bolyen et al., 2019) because of its modular design and robust quality control features that ensure reproducibility and transparency in microbiome data analysis. Raw sequences were processed using the DADA2 plugin in QIIME2 to remove low-quality reads and chimeras and generate a table of amplicon sequence variation (ASV) features. A feature classifier plugin based on the Navier Bayes classifier and the Greengenes 13_8 database was used to classify and annotate the ASVs. Also for differential abundance analyses, we used the edgeR package (Robinson and Oshlack, 2010) because of its ability to handle sparse data and provide robust statistical tests. Raw ASV counts were normalized using the TMM method in edgeR to account for differences in sequencing depth. Differential abundance was determined using a negative binomial distribution model and then corrected for multiple assumptions using the Benjamini-Hochberg method. ASVs with a false discovery rate (FDR) < 0.05 were considered significantly different between groups.

The WGCNA network was applied to divide co-expressed prefrontal gene modules, explore the correlation between gene modules and behavioral performance, and identify the key genes and highly related gene modules. Firstly, in order to construct a weighted co expression network of genes, we determined the soft thresholding powers value using the “pickSoftThreshold” function in the WGCNA package. Subsequently, based on the gene expression value matrix, the similarity of gene expression is calculated to obtain the adjacency matrix. Finally, calculate the topological overlap matrix (TOM). All genes can be classified into different clusters based on their co expression similarity patterns. There is a high degree of co expression similarity among genes in each cluster. After constructing the WGCNA co expression modules, we linked these modules to the behavioral performance and antioxidant levels of mice to explore key genes that may be closely related to cognitive function. In the joint analysis of gut microbiota and prefrontal genes expression, the first step is to compare the consistency of data changes between the two omics shape distributions through Procrustes analysis. Then spearman correlation was used to calculate the degree of association (*r* > 0.8 and *p* < 0.01) between the selected key abundant and rare microorganisms and genes with significant inter group differences, and their network diagrams were visualized. Finally, we conducted GO enrichment analysis on key genes associated with different types of data to observe their biological functions.

## Results

### Behavioral tests

Figure 2 shows the results of the behavioral tests for the memory abilities of the mice. The novel object recognition test serves as a starting point for the assessment of cognitive function and allows for a preliminary assessment of an animal’s working memory capacity and adaptation to a new environment. As shown in Figure 2B, the exploration ratio in the NOR test were increased in the P group compared with the Control group, suggesting that *Lactobacillus johnsonii* HL79 treatment may be effective in improving cognitive function. Mice in the HA group showed a significantly lower frequency of exploration toward the novel object, reflecting the fact that prolonged exposure to high altitude may have led to a decline in memory performance in mice (*p* < 0.05). Conversely, Although the frequency of exploring new objects was still lower in the HAP group than in the P group (*p* < 0.05), it was significantly higher than in the HA group (*p* < 0.05), further supporting the hypothesis that *Lactobacillus johnsonii* HL79 helps restore cognitive function impaired by long-term high-altitude exposure.

After assessing basic memory and recognition abilities, the elevated plus maze experiment allows further exploration of anxiety-like behaviors, learning and memory capacity in mice. As shown in Figures 2D,E, compared with the Control group, mice in the P group showed increased frequency of open-arm exploration and longer time into the open arm, suggesting that probiotics may have reduced the anxiety level of the mice to some extent and enhanced their exploratory behavior in the open arm. In contrast, the HA group showed a significantly lower frequency of open-arm exploration (*p* < 0.05) and less time in the open arm (*p* < 0.05), which may be attributed to the hypoxic and low-pressure conditions in the high altitude environment, which led to increased anxiety and inhibited exploratory behaviors in the mice. In contrast, the frequency of open-arm exploration and the duration of stay in the HAP group were significantly higher than those in the HA group (*p* < 0.05 and *p* < 0.01, respectively), which suggests that probiotics can alleviate the negative effects of the high altitude environment on the anxiety behaviors of mice to a certain extent.

### Antioxidant capacity in serum and whole brain tissue

**Figure 3:**
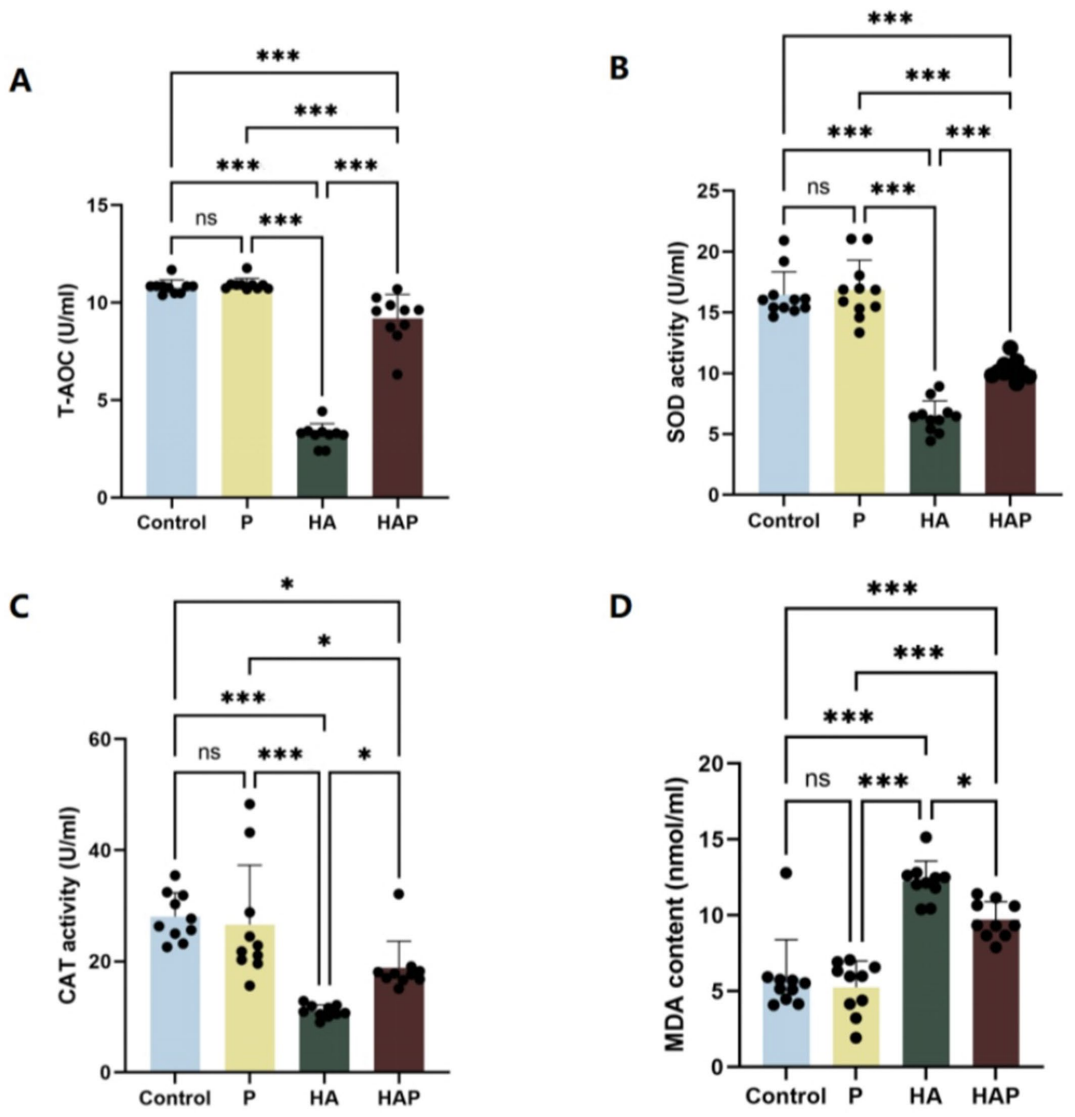
Antioxidant capacity in **(A–D)** the serum. Data are presented with the means ± standard deviation (*n* = 10). NS, not significant (*p* > 0.05); *difference is significant at the 0.05 level (*p* < 0.05); **difference is significant at the 0.01 level (*p* < 0.01); ***difference is significant at the 0.001 level (*p* < 0.001). Activities or contents of T-AOC, SOD, CAT, and MDA, respectively. T-AOC, total antioxidation capacity; SOD, superoxide dismutase; CAT, catalase; MDA, malondialdehyde.

To assess the effect of *Lactobacillus johnsonii* HL79 on oxidative stress, we measured antioxidant indices in serum and whole brain of mice. Figures 3A–D shows the antioxidant indices of serum. In terms of total antioxidant capacity (T-AOC), as shown in Figure 3A, T-AOC content increased in the P group compared to the Control group, while T-AOC content was significantly lower in the HA group (*p* < 0.001), suggesting that the high altitude environment may reduce the antioxidant capacity of mice. The T-AOC content in the HAP group was significantly higher than that in the HA group (*p* < 0.001), which indicates that the *Lactobacillus johnsonii* HL79 supplementation significantly improved this index. In terms of superoxide dismutase (SOD) activity (Figure 3B), SOD activity was significantly lower (*p* < 0.001) in the HA group compared with the Control group, which may reflect the negative impact of the high altitude environment on the antioxidant defense system of mice. However, there was a significant increase (*p* < 0.001) in SOD activity in the HAP group compared with the HA group, suggesting that *Lactobacillus johnsonii* HL79 supplementation may help to alleviate the inhibitory effect of the high altitude environment on SOD activity in mice. CAT activity, as shown in Figure 3C, CAT activity in the P group was elevated compared to the other two groups although it was not significantly different from the Control group, CAT activity in the HA group was significantly lower than that in the Control group (*p* < 0.001), and there was a significant increase in CAT activity in the HAP group compared with the HA group (*p* < 0.05). MDA content, as an indicator of oxidative stress, as shown in Figure 3D, MDA content decreased in the P group compared to the Control group, was significantly higher in the HA group compared to the Control group (*p* < 0.001), and was significantly lower in the HAP group compared to the HA group (*p* < 0.05), which suggests that probiotic supplementation may help to reduce the oxidative stress caused by the high altitude environment.

**Figure 4:**
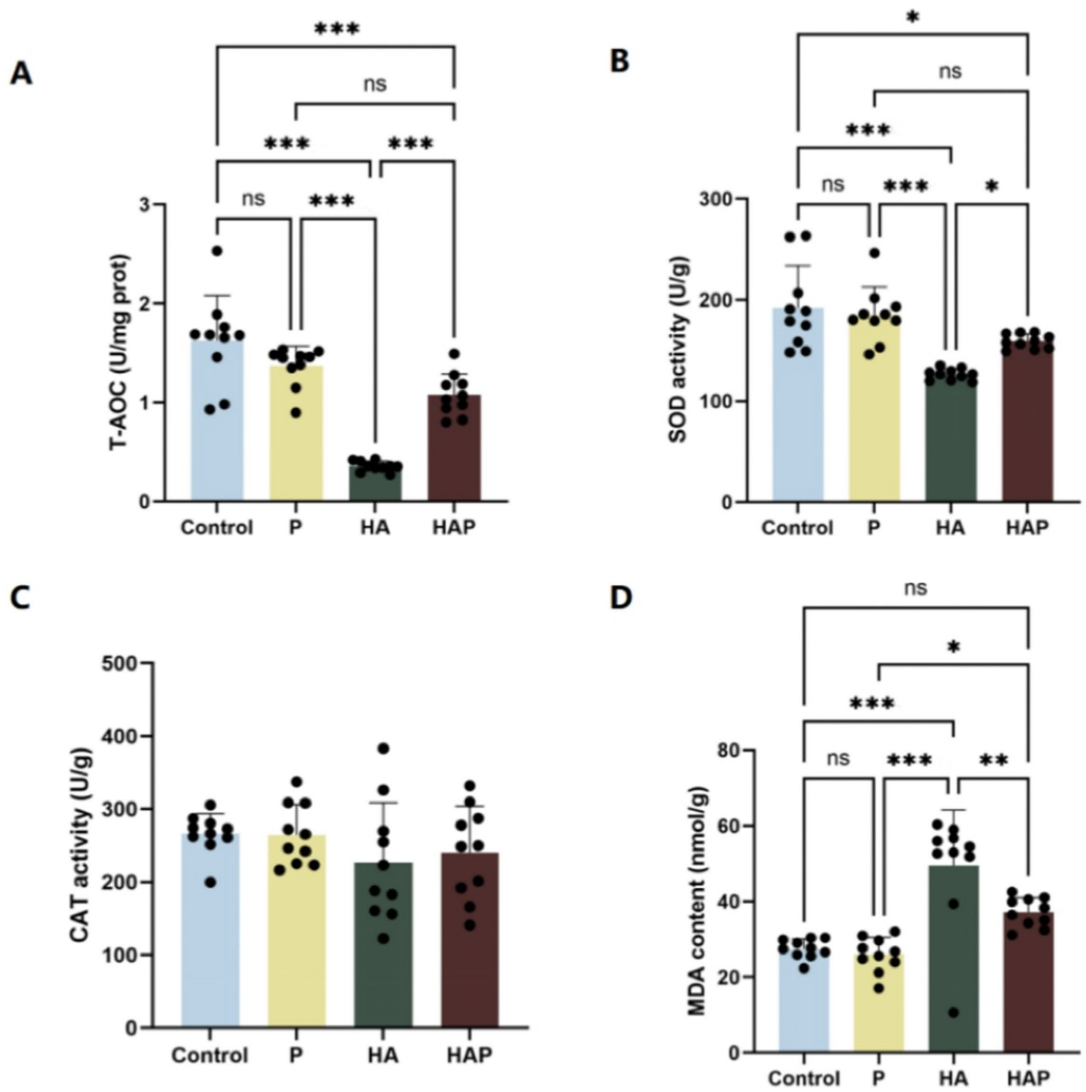
Antioxidant capacity in **(A–D)** the whole brain. Data are presented with the means ± standard deviation (*n* = 10). NS, not significant (*p* > 0.05); *difference is significant at the 0.05 level (*p* < 0.05); **difference is significant at the 0.01 level (*p* < 0.01); ***difference is significant at the 0.001 level (*p* < 0.001). Activities or contents of T-AOC, SOD, CAT, and MDA, respectively. T-AOC, total antioxidation capacity; SOD, superoxide dismutase; CAT, catalase; MDA, malondialdehyde.

Figures 4A–D demonstrates the antioxidant indices of the whole brain. In terms of total antioxidant capacity (T-AOC), as shown in Figure 4A, T-AOC content was significantly decreased in the HA group compared to the Control group (*p* < 0.001). The HAP group had significantly higher T-AOC content than the HA group (*p* < 0.001). In terms of superoxide dismutase (SOD) activity (Figure 4B), SOD activity was significantly lower in the HA group compared to the Control group (*p* < 0.001). However, there was a significant increase in SOD activity in the HAP group compared with the HA group (*p* < 0.05), suggesting that probiotic supplementation may help to alleviate the inhibitory effect of the plateau environment on whole-brain SOD activity in mice. MDA content, as shown in Figure 4C, decreased in the P group compared with the Control group, and was significantly higher in the HA group compared with the Control group (*p* < 0.001), whereas it was significantly lower in the HAP group compared with the HA group (*p* < 0.01), suggesting that the high altitude environment may reduce the antioxidant capacity of the whole brain of mice, and the *Lactobacillus johnsonii* HL79 supplementation may contribute to the reduction of whole brain oxidative stress induced by high altitude environment. In addition, as shown in Figure 4D, CAT activity was not significantly different among the four groups (*p* > 0.05).

### Effects of high-altitude environment and *Lactobacillus johnsonii* HL79 intervention on the structure of cecum microbial community in mice

**Figure 5:**
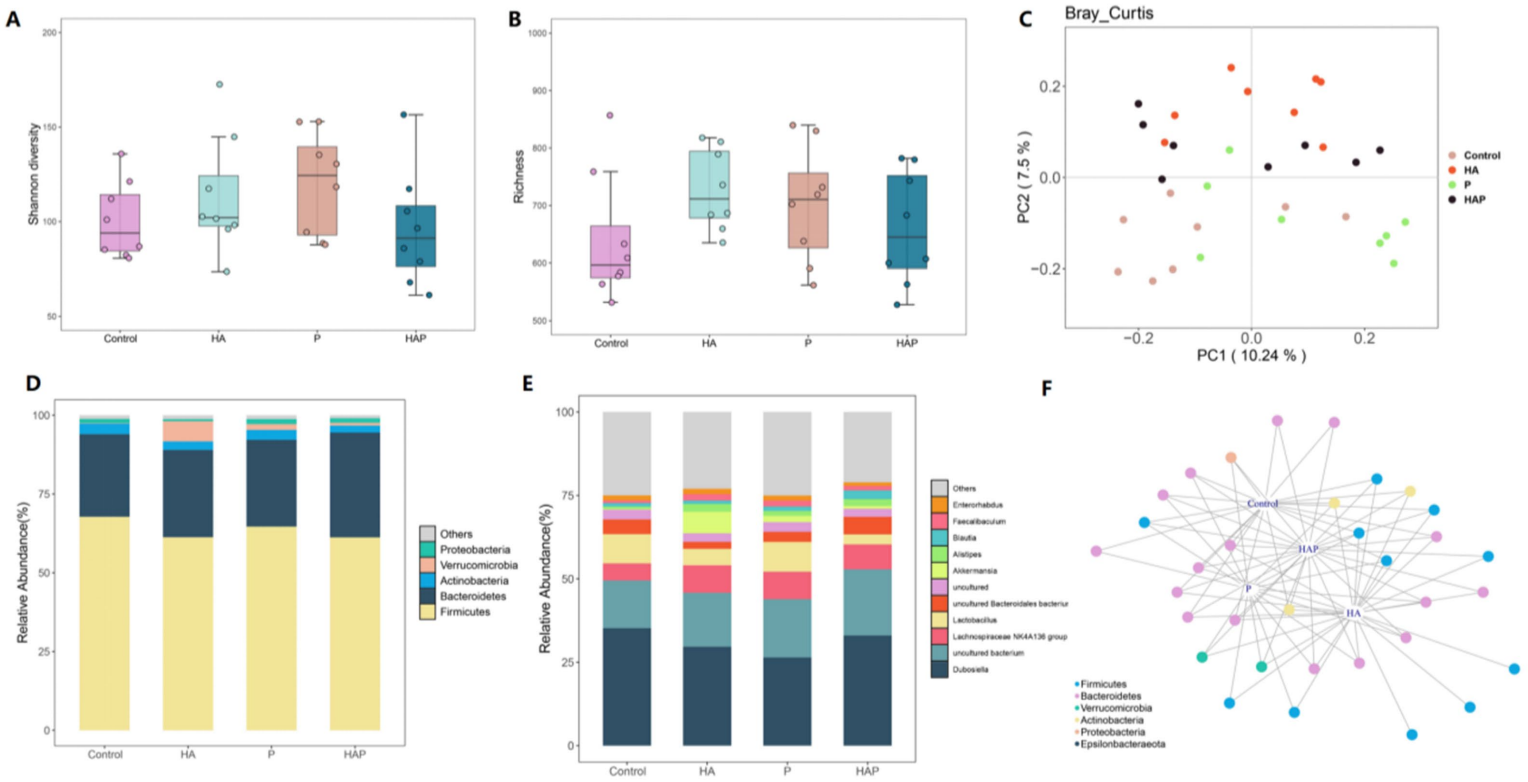
Effects of hypoxia on the structure of colon microbiota in normal mice and antibiotic drinking mice. **(A)** Intestinal microbial community diversity in each group (Shannon). Meaning: Wilcoxon. **(B)** Gut microbiome richness (observed species) in colonic luminal samples of each group. Significance: Wilcoxon. **(C)** Principal Coordinate Analysis (PCoA) of Unweighted UniFrac Distance between groups. **(D)** Relative abundance at phylum level in each group (%). **(E)** Relative abundance at each family level (%). **(F)** Networks of indicator species analysis. It displays different treatment-specific ASVs in the colonic bacterial communities using indicator species analysis. Circles represent individual bacteria and ASVs that are positively and significantly associated (*p* < 0.05) with one or more different grouping factors (association (s) given by connecting lines). ASVs are colored according to their Family assignment.

Differences in the Shannon diversity index of the cecum were statistically significant among all four groups (*p* < 0.01; Figure 5A), and richness analyses further revealed significant variations in the number of species between groups (*p <* 0.01; Figure 5B). Principal coordinate analysis (PCoA) based on the Bray-Curtis distance revealed differences in spatial distribution among samples, with the first principal component explaining 10.24% of the variation and the second principal component explaining 7.5% of the variation (Figure 5C). On the first principal coordinate (PC1), the HA group showed significant separation compared to the Control, P and HAP groups, suggesting that the high altitude environment is the main source of variation in the cecum microbiota of mice. In addition, the second principal coordinate (PC2) also showed significant separation in all four groups. The results suggest that HL79 treatment is the second major source of variation in microbial community structure. Five bacterial phyla (Figure 5D) were identified as dominant species in the cecum samples: Proteobacteria, Verrucomicrobia, Actinobacteria, Bacteroidetes, and Firmicutes. Of these, Firmicutes was the dominant species. Bacteroidetes as the second dominant species and Verrucomicrobia as the third dominant species. At the genus level (Figure 5E), we found that the genera Enterorhabdus, Faecalibaculum, and Blautia exhibited significant differences in relative abundance across the four sample groups (Control, HA, P, and HAP), and these genera may represent key microbial taxa in each sample group. Specifically, the relative abundance of Enterorhabdus in group Control may be higher than the other groups, whereas the relative abundance of Enterorhabdus and Faecalibaculum was higher in group HA, which may be indicative of the ecological roles or adaptations of these genera in high-altitude environments. There was a difference in the relative abundance of Blautia and Akkermansia in the P and HAP groups, with higher abundance of these genera in the HAP group, which may be related to the treatment of samples from this group with *Lactobacillus johnsonii* HL79. In addition, we conducted indicator species analysis and after clustering the indicator species at the phylum level, we identified six key bacterial phyla from the four groups of samples (Figure 5F), which were Firmicutes, Bacteroidetes, Verrucomicrobia, Actinobacteria, Proteobacteria and Epsilonbacteraeota. In this case, we could found that the distribution of the HAP and P groups showed that the distribution of the indicator species was closer to the control group than the HA group.

### Analysis of microbial community-specific distribution and occupancy and its identification of key nodes under different conditions

**Figure 6:**
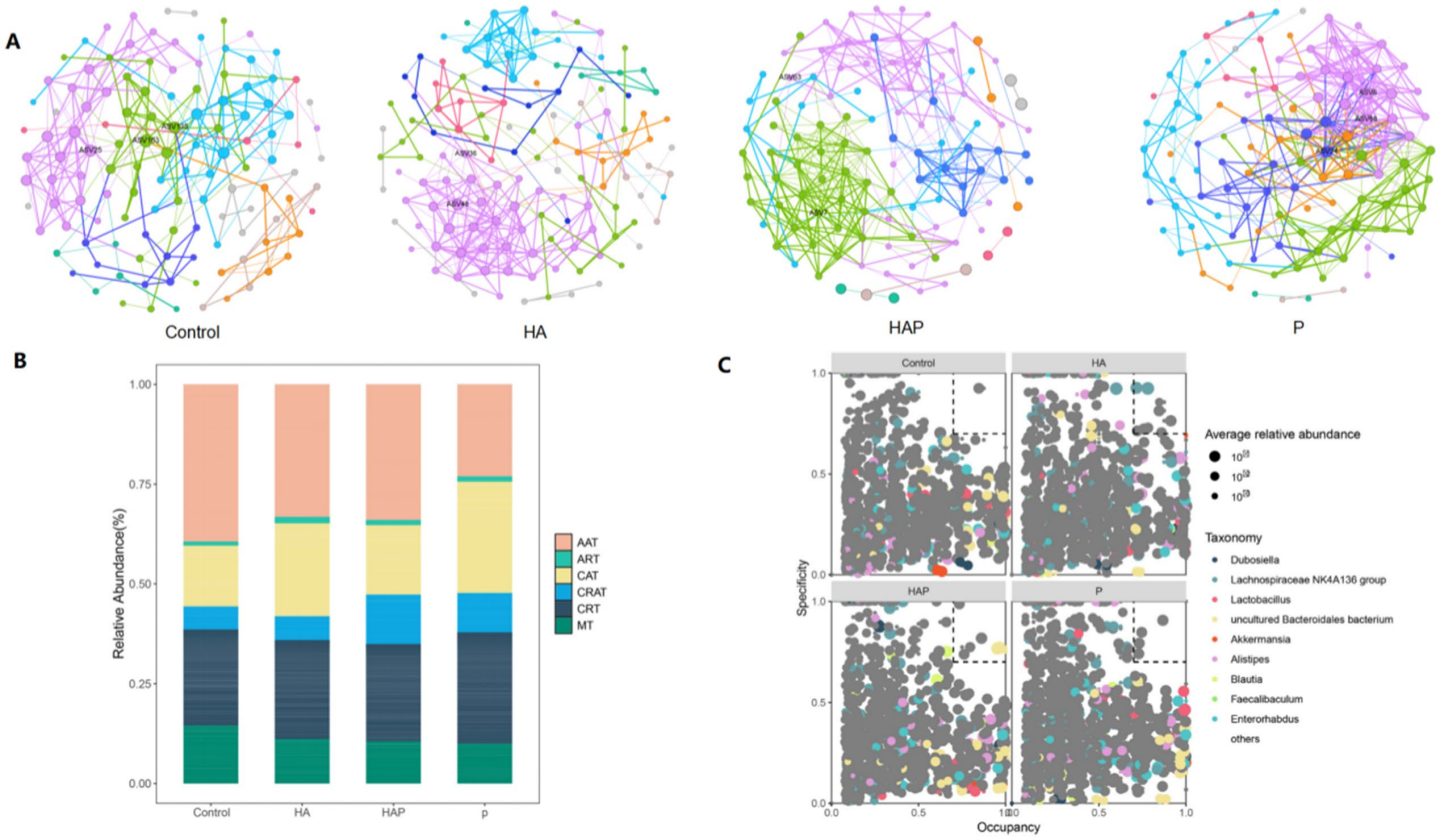
**(A)** Enriched and rare taxa. **(B)** Key species filtering within and between modules (C group, HA group, HAP group, and P group, respectively). **(C)** MENA network visualization of the C, HA, HAP, and P groups.

After an in-depth analysis of the composition of the microbial community, we identified a total of six major microbial taxa: AAT, ART, CAT, CRAT, CRT, and MT, which exhibited significant specific distribution and occupancy under different conditions (Figure 6A). Among them, species with high specificity and high occupancy were most abundant in Group Control, with most belonging to the AAT taxon, followed by a few belonging to the ART taxon; in Group HA, taxa with high specificity and high occupancy included CAT (ASV9), CRAT (ASV872 and ASV874), CRT (ASV782), and MT (ASV804); and in the HAP group, the high specific occupancy taxa included CAT (ASV17, ASV25, ASV170); in the S cluster, species with high specific occupancy were all CAT taxa (ASV17, ASV25, ASV170). Based on the specific distribution and occupancy of ASVs in different environments, we identified a total of four major bacterial genera, including *Dubosiella, Lachnospiraceae NK4A136 group*, *Lactobacillus*, and *uncultured Bacteroidales bacterium* (Figure 6B). Among them, in group Control, ASV412 showed high specificity and high occupancy; in group HA, genera with high specificity and occupancy included *Lachnospiraceae NK4A136 group* (ASV44 and ASV120), and Romboutsia (ASV203, ASV296, and ASV304). In addition, *Oscillibacter* (ASV443) and *uncultured bacterium* (ASV422 and ASV1343) also showed high specificity and occupancy; genera with high specific occupancy in the HAP group included *uncultured Bacteroidales bacterium* (ASV45, ASV50, ASV70, and ASV80), and *Clostridium sensu stricto 1* (ASV230 and ASV371). In addition, *Marvinbryantia* (ASV382) and *Anaeroplasma* (ASV596) also showed high specificity and occupancy in this group; in group P, *Bifidobacterium* (ASV292), *Roseburia* (ASV308) and *Escherichia-Shigella* (ASV317) showed high specificity and occupancy, *uncultured bacterium* (ASV333, ASV429) also showed high occupancy in this group.

Through MENA network analysis (Figure 6C), we successfully identified key microbial nodes in the four groups of mutualistic networks. In group Control, the maximum degree node was ASV163 (*uncultured Bacteroidetes*), the maximum interdependence and maximum stress centrality node was ASV135 (*Bifidobacterium*), and the maximum eigenvector centrality node was ASV25 (*Uncultured Bacteria*); in group HA, the maximum degree and maximum eigenvector centrality nodes were ASV48 (*Lachnospiraceae NK4A136 group*), and the maximum interdegree and maximum stress centrality node was ASV36 (*Toricibacter*); in the HAP group, the maximum degree and maximum eigenvector centrality node was ASV7 (*uncultured bacterium*), and the maximum interdegree and maximum stress centrality node was ASV63; in the P group, the maximum degree nodes were ASV8 (*uncultured bacterium*) and ASV68 (*uncultured bacillus-like organisms*) In group P, the maximum inter-degree and maximum eigenvector centrality nodes were ASV68 (*uncultured Bacteroidetes*) and the maximum stress centrality node was ASV24.

### Identifying differentially expressed genes in the frontal lobe and multi-omics joint analysis

**Figure 7:**
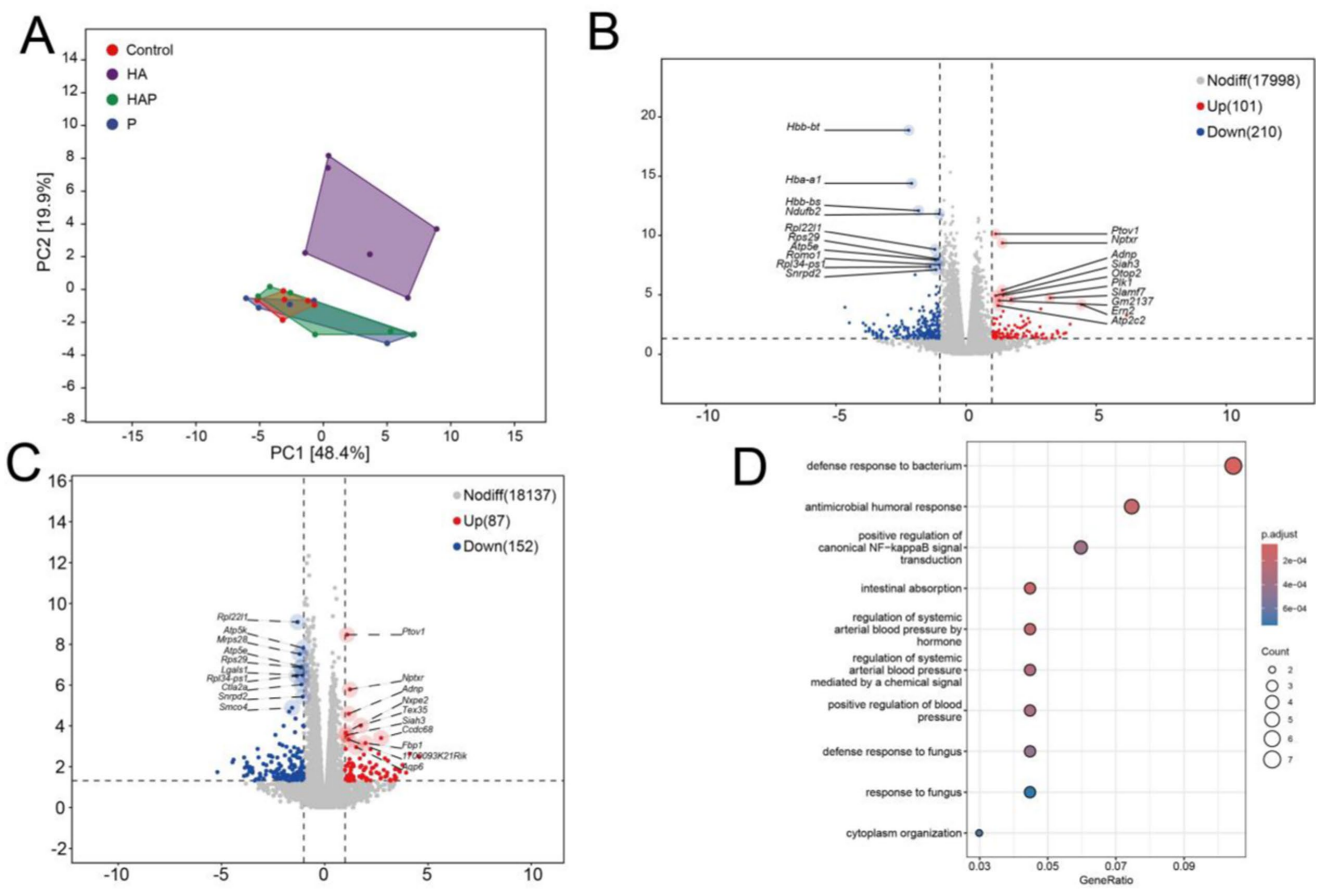
**(A)** Principal components analysis (PCA). PCA analysis can cluster similar samples together, and the closer the distance, the higher the similarity between samples. Draw volcano maps based on differentially expressed genes between **(B)** C group and HA group, as well as between **(C)** HA group and HAP group. Volcanic maps are based on expression fold differences and significance results, displaying the distribution of genes. **(D)** GO enrichment bubble chart. After completing the differential gene analysis, classify the genes with differential expression between groups based on the annotation information of the genome, and analyze the biological functions involved by these genes.

To explore the potential influence by which *Lactobacillus johnsonii* HL79 alleviates the cognitive impairment in the brain, we performed the transcriptomic sequencing to verify differentially expressed gene in frontal lobe exposed to high altitude environment and treated with *Lactobacillus johnsonii* HL79. Firstly, principle component analysis (PCA) was conducted to quantify the expression levels in each sample (Figure 7A). Notably, HA and other three groups were obviously separated, indicating a significant influence in the transcriptome profile. According to the gene data in each sample, the differential expression was showed by edgeR method. Volcano results indicated that a total of 311 and 239 differentially expressed gene were identified between Control group and HA group, and between HA group and HAP group, respectively (Figures 7B,C).

Then we used the Gene Ontology database to enrich and analyze the biological significance of the target gene and their related biological functions. Figure 7D indicates the GO enrichments based on co-differential expressed genes with the lowest false discovery rate value among different groups. GO terms, which the co-differentially expressed genes, were mainly enriched top 10 in defense response to bacterium, intestinal absorption, antimicrobial humoral response, regulation of systemic arterial blood pressure by hormone, regulation of systemic arterial blood pressure mediated by a chemical signal, positive regulation of blood pressure, positive regulation of canonical NF-kappaB signal transduction, defense response to fungus, cytoplasm organization, response to fungus.

**Figure 8:**
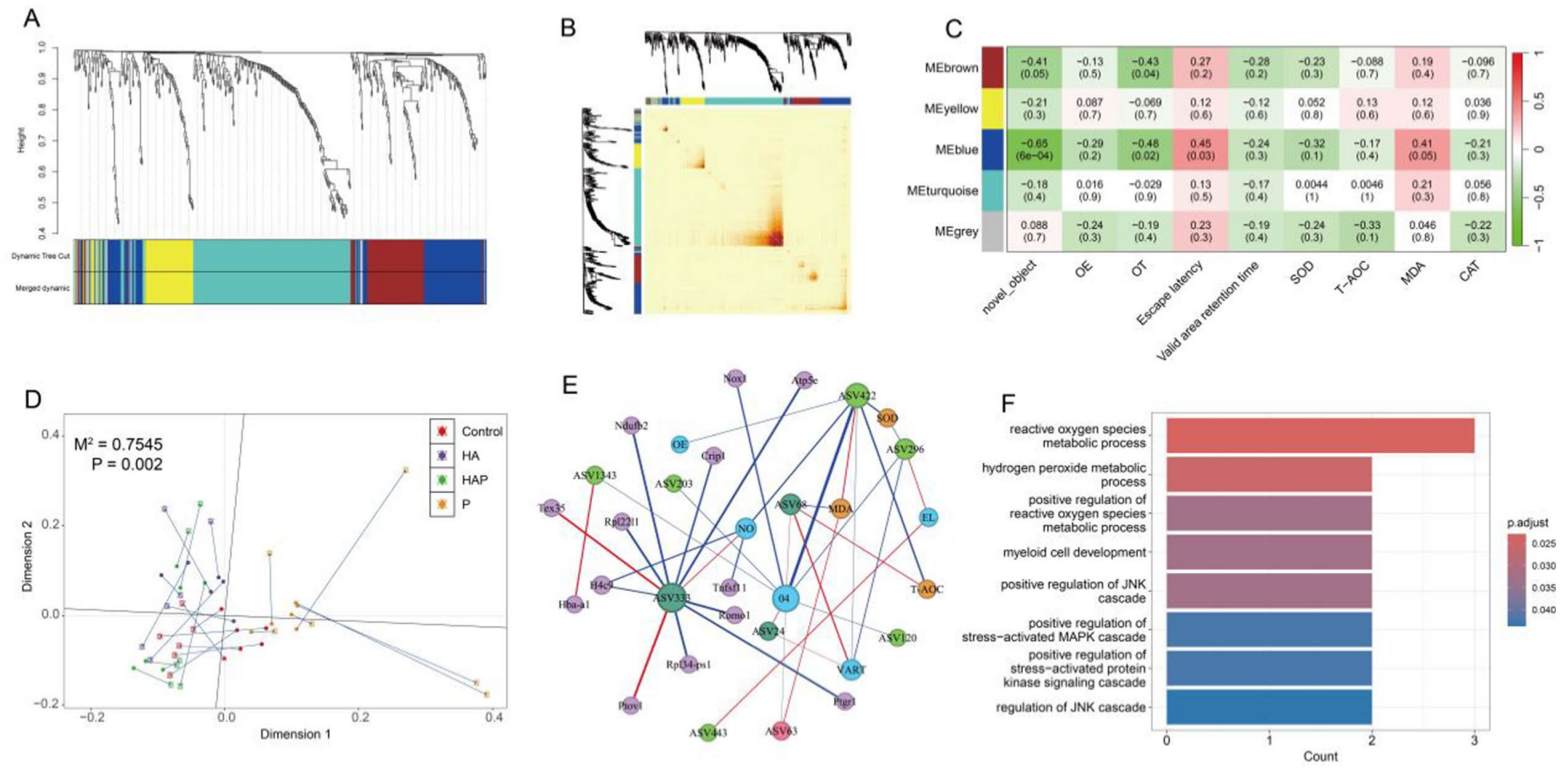
Module clustering of all DEGs based on different metrics **(A)** tree diagram and **(B)** heatmap. **(C)** Correlation heatmap of modules related to behavioral performance and antioxidant outcomes (with correlation coefficients and *p*-values for each cell). **(D)** The M2 statistical measures analyzed by Procrustes and the significant *p*-values obtained based on 999 permutation tests indicate a strong correlation between the bacterial species abundance composition and transcriptome gene abundance composition of each sample. **(E)** Calculate the correlation between variables based on three matrices (microbial abundance matrix, gene abundance matrix, and clinical detection matrix), and finally integrate all the correlation relationships to construct a network. **(F)** GO enrichment bar chart. After completing the multi-omics association analysis, the differentially expressed genes between groups were classified based on the annotation information of the genome, and the biological functions involved in these genes were analyzed.

In WGCNA analysis, the soft thresholding powers was 4 (*R*^2^ = 0.85), and 5 modules were ultimately found after dynamic tree trimming and average hierarchical clustering (Figure 8A). And the heatmap reflected the expression of similarity between different genes increased with the depth of color (Figure 8B). Above results proved that the genes in the same module have a higher common expression pattern, while the difference between different module was obvious. Figure 8C showed network’s modules have associated with the behavioral performance and antioxidant levels of frontal lobe. We observed brown and blue modules were identified to significant correlation with the changes of new object recognization, OT% of the high cross maze, escape latency of water maze and MDA content. For filtering module genes, we determined the key genes of blue module under following conditions: *p* < 0.05, correlation with behavioral results |cor| > 0.05 and no_gray module.

Based on the previous data, we performed Procrustes analysis using two omics PCA coordinates to characterize the potential consistency of gut species abundance composition and prefrontal gene abundance in each sample. As shown in Figure 8D, the potential relationship between gut microbiota and prefrontal gene expression showed excellent consistency (*p* = 0.002). Through the correlation network between different omics and physiological indicators, it can be found that the P group has the highest number of key genes related to the key species ASV333 (Muribaculaceae). In addition, key species ASV422 (Muribaculaceae) and ASV296 (Romboutsia) in the HA group are strongly correlated with multiple behavioral and antioxidant indicators, but this interaction is not related to genes in the prefrontal cortex (Figure 8E). GO enrichment analysis of genes within correlation network revealed that top 8 GO terms were significantly enriched in reactive oxygen species metabolic process, hydrogen peroxide metabolic process, positive regulation of reactive oxygen species metabolic process, myeloid cell development, positive regulation of JNK cascade, positive regulation of stress-activated MAPK cascade, positive regulation of stress-activated protein kinase signaling cascade, regulation of JNK cascade (Figure 8F).

## Discussion

Chronic exposure to high-altitude environments leads to insufficient oxygen supply and increased stress in the brain, which affects cognitive functions, including memory loss, poor concentration, and decreased decision-making ability (Bao et al., 2023). In this study, we assessed the effects of long-term high-altitude environmental exposure on cognitive functions in mice and the intervention effect of *Lactobacillus johnsonii* HL79 by behavioral tests. The novel object recognition test is a behavioral test that uses the natural tendency of rodents to explore novelty to assess their working memory and recognition memory abilities, and this test is commonly used as a starting point for the assessment of cognitive function (Ennaceur and Delacour, 1988). According to the results of the behavioral test in the present study, mice in the HA group had a significantly lower rate of exploration than those in the Control group, indicating impaired working memory capacity. This result is consistent with previous studies suggesting that high-altitude environments lead to cognitive decline, especially in working memory (Zhao et al., 2022). In addition, the exploration rate of mice in the HAP group was significantly higher than that of the HA group, suggesting that *Lactobacillus johnsonii* HL79 was able to restore, to some extent, the working memory capacity impaired by prolonged exposure to high altitude, thus revealing that the gut microbiota is closely related to brain function, and that an imbalance of which may lead to impaired gut barrier function, which in turn may affect brain function and cognitive ability (Montalbán-Rodríguez et al., 2024), and the gut microbiota may in turn affect the cognitive function and emotional state of the host through the gut-brain axis (Suda and Matsuda, 2022). Based on the above research, the elevated cross maze experiment could further explore anxiety-like behavior, learning and memory capacity in animals. This experiment can provide information on how animals deal with potential threats and explore unknown environments, which is associated with decision-making and risk assessment in cognitive functioning (Lister, 1987). Mice in the HA group had higher levels of anxiety as evidenced by a reduced frequency of exploration of the open arm and dwell time, which was associated with hypoxic and low-pressure conditions; whereas *Lactobacillus johnsonii* HL79 supplementation likewise significantly increased the frequency of exploration of the open arm and dwell time, which may affect brain function and reduce anxiety levels by modulating the gut microbiota, a result consistent with Ma et al.’s report that probiotics may alleviate symptoms of stress and anxiety by modulating the neuroactivity of the gut flora (2021). The results of the behavioral tests provide new evidence that brain dysfunction induced by prolonged altitude exposure may be closely related to the microbiota-gut-brain axis (MGBA). One study found that the degree of cognitive impairment in long-term plateau migrants was significantly correlated with the duration of migration. In addition, the low-pressure hypoxic environment triggers intestinal barrier damage, leading to lipopolysaccharide leakage and inflammatory factor influx into the peripheral blood, which ultimately leads to microglia activation and neuronal damage in the brain (Zhou et al., 2024). In this study, *Lactobacillus johnsonii* HL79, as a probiotic, may enhance the normal function of the gut-brain axis by regulating the composition and function of the intestinal microbiota, thus ameliorating the negative effects of the high-altitude environment on cognitive function.

Detecting antioxidant levels in blood and whole brain tissue is an important method to assess the body’s and brain’s response to oxidative stress. Antioxidant indices in the blood reflect the overall oxidative stress status of the organism (Sies, 1993), whereas antioxidant levels in whole brain tissues are directly linked to protective mechanisms of brain function (Sies et al., 2017). Oxidative stress is one of the key factors contributing to a variety of neurodegenerative diseases and cognitive dysfunctions, therefore, understanding the effects of high-altitude environments on these antioxidant metrics is crucial to unraveling their potential impact on cognitive function (Wang et al., 2017). In high-altitude environments, the body’s level of oxidative stress is significantly elevated due to insufficient oxygen supply and reduced barometric pressure, leading to a decrease in antioxidant enzyme activity and an increase in oxidation products (Pena et al., 2022). This oxidative stress not only affects physiological functions throughout the body, but also adversely affects cognitive functions in the brain, especially in brain regions that are closely related to cognitive functions, such as the prefrontal cortex (Fan et al., 2024). Meanwhile, it has also been found that oxidative stress can lead to cognitive decline by inducing neuroinflammation and damaging hippocampal structures (Zhang et al., 2024). In addition, genetic variants of antioxidant enzymes were significantly associated with cognitive enhancement, suggesting that antioxidant enzyme activity may have a protective effect on cognitive function (Wang et al., 2022). It has also been shown that cognitive dysfunction can be improved by modulating the level of oxidative stress. For example, green tea extract showed a protective effect on cognitive function in middle-aged and elderly people by reducing oxidative stress and improving cognitive function (Fan et al., 2024). This suggests that oxidative stress negatively affects cognitive function through mechanisms such as inducing neuroinflammation, damaging hippocampal structure, and affecting antioxidant enzyme activity, and that modulating oxidative stress levels may offer a potential avenue of intervention to improve cognitive dysfunction. Therefore, we investigated antioxidant indices in blood and whole brain tissues of mice to determine the mechanisms by which chronic altitude exposure stress induces cognitive dysfunction in mice and how *Lactobacillus johnsonii* HL79 protects the host from cognitive dysfunction. High altitude environments result in inadequate oxygen supply and reduced barometric pressure, which increase the body’s level of oxidative stress, decrease T-AOC and weaken antioxidant defenses (Pena et al., 2022). In high altitude environments, impaired function of the mitochondrial respiratory chain leads to increased production of superoxide anion radicals, which cannot be scavenged in a timely manner due to reduced SOD activity, exacerbating oxidative stress; at the same time, the accumulation of hydrogen peroxide with reduced CAT activity prevents its effective breakdown, increasing the risk of oxidative damage to cells (Srivastava et al., 2017; Denu and Hematti, 2021). In addition, increased MDA content reflects the extent of oxidative damage to cell membrane lipids (Tsikas, 2017). In our study, prolonged high altitude exposure significantly reduced total antioxidant capacity (T-AOC), superoxide dismutase (SOD) and catalase (CAT) activities in the blood of mice, while increasing malondialdehyde (MDA) content, suggesting that high altitude environments trigger oxidative stress (Janocha et al., 2017). Notably, whole brain tissue showed similar trends in antioxidant metrics, although these changes have not yet reached statistical significance. These changes in oxidative stress may adversely affect brain function. In contrast, *Lactobacillus johnsonii* HL79 supplementation significantly increased T-AOC, SOD and CAT activities and decreased MDA content in high altitude exposed mice, suggesting that it is effective in alleviating oxidative stress induced by high altitude environment (Liu et al., 2024). Thus, our findings clearly indicate that enhanced oxidative stress is at least partially related to cognitive dysfunction induced by chronic high altitude exposure, and that probiotic supplementation can effectively alleviate oxidative stress induced by high altitude environments through the microbiota-gut-brain axis, a result that is consistent with that reported by Burokas et al. (2015).

Previous studies have found that cognitive dysfunction, especially the decline in working memory, is mediated by the gut microbiota in high-altitude environments, implying that the gut microbial structure is closely related to the hypoxic environment (Li et al., 2024; Su et al., 2024). In the present study, we performed a differential analysis of the mouse cecum microbial community to reveal the differential expression of microbial taxa under high-altitude environment and probiotic treatment. Our results showed statistically significant differences in Shannon diversity index and species richness among the four groups of mice, suggesting that environmental and probiotic factors have a significant effect on microbial diversity (Keck and Kahlert, 2019). Using principal coordinate analysis (PCoA) based on the Bray-Curtis distance, we further revealed differences in spatial distribution among the samples. The significant clustering of the HA group on PC1 compared to the C, P and HAP groups highlights the profound effect of high-altitude conditions on the structure of microbial communities. In addition, the second principal coordinate (PC2) also showed significant separation in all four groups, suggesting that probiotic treatments played an important role in distinguishing the microbial community structure of the different groups. Five dominant phyla identified in our mouse cecum samples - Proteobacteria, Verrucomicrobia, Actinobacteria (Actinobacteria), Bacteroidetes, and Firmicutes - revealed the core microbial composition. Changes in the distribution and abundance of these phyla provide important information for our understanding of microbial community adaptations in different environments (Pral et al., 2021). At the genus level, the genera *Dubosiella*, *Lachnospiraceae NK4A136 group, Lactobacillus,* and *uncultured Bacteroidales bacterium* showed significant differences in relative abundance among the four groups. The relative abundance of *Dubosiella* in group C may be higher than that of the other groups, whereas the relative abundance of *Lachnospiraceae NK4A136 group* and *Bifidobacterium* was higher in group HA which may be indicative of the ecological roles or adaptations of these genera in high altitude environments (Liu et al., 2024). Differences in the relative abundance of *uncultured Bacteroidales bacterium* and *Akkermansia* between the P and HAP groups, with a higher abundance of these genera in the HAP group, may be related to the treatment of samples from this group with *Lactobacillus johannesii* (Wan et al., 2022). Further through indicator species analysis, we identified six key bacterial phyla from the four groups of samples, which were Firmicutes, Bacteroidetes, Verrucomicrobia, Actinobacteria, Proteobacteria and Epsilonbacteraeota. The relative abundance of Firmicutes was significantly higher in Group C than in Group HA, an observation that may indicate the high sensitivity of Firmicutes to changes in environmental parameters (Filippidou et al., 2016). In the comparative analysis between P and HAP groups, Bacteroidetes showed a more significant change in abundance in the HAP group compared to the P group, a change that may indicate the enhanced responsiveness of *Bacteroidetes* to the intervention of *Lactobacillus johannesii* HL79.

In previous studies, rare microorganisms have often been considered as confounding factors and have been further eliminated. However, in the present study we found a high relative percentage of both rare and abundant taxa in the cecum. Thus, rare species may also be potentially responsible for important effects on the plateau environment and *Lactobacillus johannesii* HL79.

In this study, we used MENA network analysis to reveal a complex map of abundant microbial community composition, showing the specificity and occupancy of key microbial species under different environmental conditions. Our findings complement the body of knowledge initiated by Matchado et al. (2021), highlighting the complex interactions between microbial communities and their ecological niches. In previous studies, rare microorganisms were usually considered as interfering factors and further eliminated. But in this study, we found that the relative proportion of rare and abundant taxa in the cecum was relatively high. Therefore, rare species may also be a potential reason for the significant impact on high-altitude environments and *Lactobacillus johnsonii* HL79. The six major microbial groups we identified - AAT, ART, CAT, CRAT, CRT, and MT - demonstrate a subtle distribution of microbial species with high specificity and occupancy, particularly in group C, which is rich in AAT and ART species. This observation is consistent with the ecological theory that specific environmental conditions select for certain microbial taxa, e.g., taxa with high specificity and occupancy in the HA group include CAT (ASV9), CRAT (ASV872 and ASV874), CRT (ASV782), and MT (ASV804). The high specificity and occupancy observed in specific bacterial genera, such as *Dubosiella*, *Lachnospiraceae NK4A136 group*, *Lactobacillus*, and u*ncultured Bacteroidales bacteria*, provide a deeper understanding of the taxonomic composition and ecological significance of these microorganisms. In particular, species ASV44 and ASV120 in the HA group and ASV45, ASV50, ASV70 and ASV80 in the HAP group were identified as potential keystone species, emphasizing their importance in microbial community structure and function. In addition, MENA network analyses identified microbial nodes that may play key roles in the ecosystem. Nodes such as ASV7 (*uncultured bacteria*) and ASV63 in the HAP group showed high centrality metrics, suggesting that they may have an impact on microbial network dynamics. These findings are in line with the work of Matchado et al. (2021) and Yang et al. (2024), who emphasized the importance of culture in understanding “unculturable” human microbial communities.

To our knowledge, there have been no reports on the effects of probiotics on brain gene expression in high-altitude environments. We conducted differential analysis through transcriptomics of the prefrontal cortex and obtained differentially expressed genes under different effects of high-altitude environment and probiotics. Enrichment analysis proves that these shared differentially expressed genes are mainly involved in the biological processes of defending against bacterial and fungal infections. Use differential genes obtained from comparisons between different groups for WGCNA to identify co expressed modules related to cognitive function in the prefrontal cortex. The genes within modules in the network have high co expression similarity, suggesting that they participate in similar regulatory pathways or play a role in similar biological functions. Our research has demonstrated a close relationship between gene expression in the Blue module and behavioral performance. Therefore, subsequent analysis focused more on the intersection of these co expressed genes with differentially expressed genes between different groups, and obtained 38 genes that significantly affect cognitive function under different treatments of high altitude and probiotics. Subsequently, we conducted correlation analysis between the selected key gut species and key genes in the prefrontal cortex with behavioral and antioxidant results, and identified potential reasons for the impact on cognitive function under different treatments. There is a significant correlation between the key species in the HA group and their behavioral performance. No indirect involvement of prefrontal gene expression was observed in this process, suggesting that key species in the HA group, Muribaculaceae (ASV422, ASV1343), Romboutsia (ASV296, ASV203), *Lachnospiraceae NK4A136 group* (ASV120), and Lachnospiraceae (ASV413), may directly affect cognitive function levels through other means. Muribaculaceae, as a beneficial bacterium, has been shown to participate in the body’s anti-inflammatory response and oxidative stress processes (Yuan et al., 2025; Zeng et al., 2023), which is similar to our findings. In addition, research has found that Romboutsia is closely related to obesity, Crohn’s disease and diabetes. Meanwhile, Romboutsia is also considered to play a crucial role in regulating immune function (Luo et al., 2024; Russell et al., 2019).

The key species Muribaculaceae (ASV333) under the action of probiotics is significantly associated with more co expressed genes in the blue module. For example, Romo1, a reactive oxygen species regulatory gene significantly negatively correlated with ASV333, has been shown to be a novel mitochondrial transmembrane protein in cells that induces mitochondrial ROS production through mitochondrial electron transport chain complex III (Mendiola et al., 2023). In addition, CRIP1 has been shown to be significantly negatively correlated with ASV333 in this study, and it participates in the pathogenesis of multiple myeloma through dual regulation of proteasome and autophagy (Tang et al., 2024). Finally, genes strongly associated with key species and behavioral performance are mainly enriched in functions related to oxidative stress. In summary, we can find that there may be differences in the pathways through which high-altitude environments and probiotics affect cognitive function, and these differences are mainly manifested in the gene expression process of the prefrontal cortex.

In addition to the antioxidant capacity and modulation of the gut microbiota shown in the results above, *Lactobacillus johnsonii* HL79 may also have beneficial effects on cognitive function through other mechanisms. For example, previous studies have shown that certain probiotics can reduce neuroinflammation by modulating the release of pro-inflammatory cytokines and inhibiting the activation of microglia, the resident immune cells in the brain (Del Toro-Barbosa et al., 2020; Binda et al., 2024). This anti-inflammatory effect is critical in high altitude environments where neuroinflammation tends to be exacerbated by hypoxia-induced oxidative stress (Chen et al., 2023). In addition, *Lactobacillus johnsonii* may also affect levels of neurotransmitters such as serotonin, dopamine, and glutamate, which are critical for cognitive function and mood regulation (Cryan et al., 2019; Di Chiano et al., 2024). By modulating the gut-brain axis, HL79 has the potential to increase the secretion of these neurotransmitters in the brain, leading to improved cognitive performance and reduced anxiety behaviors in high altitude-exposed mice (Ma et al., 2021).

## Conclusion

The long-term exposure to high altitude exacerbates cognitive dysfunction in mice, particularly the decline in working memory, by affecting the gut microbiota-gut-brain axis (MGBA). However, administration of the probiotic *Lactobacillus johnsonii* HL79 significantly improves cognitive function by modulating the gut microbiota and antioxidant capacity, highlighting the importance of MGBA in high-altitude environments. The transcriptomic analysis of the prefrontal cortex revealed novel findings, including the identification of differentially expressed genes (DEGs) enriched in immune and neurovascular functions, as well as key gene modules associated with cognitive performance and antioxidant levels. These findings not only support the hypothesis that HL79 mitigates high-altitude-induced cognitive dysfunction through the gut-brain axis but also uncover new molecular pathways and potential therapeutic targets for cognitive enhancement in high-altitude settings. Future research should focus on validating these mechanisms and exploring the clinical potential of HL79 in mitigating cognitive decline in high-altitude environments.

Future research should focus on elucidating the detailed mechanisms underlying the beneficial effects of *Lactobacillus johnsonii* HL79, particularly its interactions with the host’s immune system, neuroinflammation pathways, and neurotransmitter regulation. Additionally, clinical trials are warranted to evaluate the efficacy and safety of HL79 in human populations exposed to high-altitude environments. This could pave the way for the development of personalized probiotic therapies tailored to mitigate cognitive decline and enhance overall well-being in high-altitude settings.

## Data Availability

The 16S rRNA sequencing and transcriptome reads data have been uploaded to NCBI. The login codes for the sequences were read from the archives of the National Center for Biotechnology Information (NCBI) Bioprojects database: PRJNA1210798 and PRJNA1210782, respectively.
